# Structure and Flexibility of the C-Ring in the Electromotor of Rotary F_o_F_1_-ATPase of Pea Chloroplasts

**DOI:** 10.1371/journal.pone.0043045

**Published:** 2012-09-25

**Authors:** Shai Saroussi, Maya Schushan, Nir Ben-Tal, Wolfgang Junge, Nathan Nelson

**Affiliations:** 1 Department of Biochemistry and Molecular Biology, George S. Wise Faculty of Life Sciences, Tel-Aviv University, Ramat Aviv, Israel; 2 Division of Biophysics, University of Osnabrück, Osnabrück, Germany; University of Hyderabad, India

## Abstract

A ring of 8–15 identical c-subunits is essential for ion-translocation by the rotary electromotor of the ubiquitous F_O_F_1_-ATPase. Here we present the crystal structure at 3.4Å resolution of the c-ring from chloroplasts of a higher plant (*Pisum sativum*), determined using a native preparation. The crystal structure was found to resemble that of an (ancestral) cyanobacterium. Using elastic network modeling to investigate the ring's eigen-modes, we found five dominant modes of motion that fell into three classes. They revealed the following deformations of the ring: (I) ellipsoidal, (II) opposite twisting of the luminal circular surface of the ring against the stromal surface, and (III) kinking of the hairpin-shaped monomers in the middle, resulting in bending/stretching of the ring. Extension of the elastic network analysis to rings of different c*_n_*-symmetry revealed the same classes of dominant modes as in *P. sativum* (c_14_). We suggest the following functional roles for these classes: The first and third classes of modes affect the interaction of the c-ring with its counterparts in F_O_, namely subunits a and bb'. These modes are likely to be involved in ion-translocation and torque generation. The second class of deformation, along with deformations of subunits γ and ε might serve to elastically buffer the torque transmission between F_O_ and F_1_.

## Introduction

ATP (adenosine tri-phosphate), the general energy currency of the cell, is supplied mainly by F_O_F_1_-ATPase (ATP synthase). This enzyme is composed of two rotary machines, F_O_ and F_1_, which are coupled by a central rotor and a peripheral stator. F_O_ translocates ions (mostly protons) and generates torque at the expense of the ion-motive force, and F_1_ synthesizes ATP at the expense of the mechanical torque provided by F_O_ [1], [2], [3], [4], [5]. The construction principles of this enzyme have been conserved across a range of species and structures spanning eubacteria, mitochondria and the chloroplasts of higher organisms. A homo-oligomeric ring of the hairpin-shaped subunit c is included in the Fo domain. The number of copies of c subunits in this ring varies across organisms and ranges from 8 (bovine mitochondria [6]) to 15 (cyanobacteria [7]). In other words, the ion-to-ATP machines of different organisms operate with different sized ‘gears’. Researchers agree, however, that in a given organism the number of copies of subunit c is constant, independent of the metabolic state [8], [9]. The FoF_1_-ATPase in chloroplasts, CFoCF_1_ [1], [10], is the subject of this work.

The c-ring of the enzyme's CFo portion consists of 14 copies of subunit c [11], [12], and is embedded in the membrane. The ring is attached to a complex consisting of subunit a and a dimer comprising two b-type subunits; the dimer connects F_o_ with F_1_ (in chloroplasts this dimer is a bb' heterodimer). Despite being bound to abb', the c-ring is able to rotate relative to these subunits. Subunit a hosts an essential arginine residue, which faces the essential glutamic acid residue situated at the center of a proximate hairpin-shaped monomer of c-subunit. Subunit a also contains two proton half-channels connecting the acidic residue on the c-subunit with the lumen and the stroma phases. Brownian fluctuations, together with the ion-driven rotation of the c-ring relative to the two half-channels on subunit a, are responsible for generating the torque associated with rotary proton translocation (see [5], [13], [14]). The stepped rotary motion of the c-ring relative to the stator has recently been resolved experimentally for the *Escherichia coli* enzyme [15], [16]. The F_1_ portion of the ATP synthase is characterized by a 3-fold stepping rotation; the mismatch between this rotation and the 8-to-15-fold stepping of the Fo-portion of the ATP synthase is buffered by an elastic power transmission between these motors. For the *E. coli* enzyme, the stiffness of the stator [17] and a great elastic compliance of the rotor have been determined (see [18], [19], [20], [21], reviewed in [5]). These functional features of the FoF_1_-ATPase are probably shared by the V-ATPase, which is characterized by similar structures [22].

**Table 1:** Data collection and refinement statistics.

***Data statistics***	
Wavelength (Å)	1.000 Å
Space group	C2
Unit cell parameters (Å,°)	a = 139.3, b = 102.54, c = 122.63 β = 101.22
Total reflections	79,289
Unique reflections	27,237
Completeness (%)	99.2 (86)*
Rpim (%)	14.5 (80) ±
I/σ	3.3 (1.0) ±
Resolution range (Å)	82–3.4
X-ray source	ESRF beam line ID23-1
***Refinement statistics***	
Resolution range (Å)	28–3.4 (3.58–3.4)±
No. of reflections (working/test)	20150/1044
dmin (Å)	3.4
Rwork/Rfree (%)	29/32 (0.39/0.45)±
RMS deviation from ideality:	
Bond lengths	0.008
Bond angles	1.2
Average B factor (Å^2^)	78
Solvent content (%)	63

* after anisotropic scaling.

± highest resolution shell.

The previously determined crystal structures of subunit c have provided important insight into the role of the c-ring in the proton translocating mechanism. In a given organism, the c-ring, composed of 8–15 monomers as noted above, encompasses an ion-binding site approximately in the ring's middle plane [6], [12], [23], [24], [25], [26], [27]. The functional acidic residue (mostly glutamate, in some organisms aspartate) is situated at the outward-facing helix of the hairpin. It is stabilized by hydrogen bonds with neighboring residues. The dynamics of the ion-binding site have been simulated by molecular dynamics (MD), revealing structural transitions at the nanosecond time scale upon glutamate deprotonation/protonation [28], [29]. However, MD simulations have been restricted to a narrow time window of 10–100 ns and have not revealed large-scale motion in micro- to milliseconds as relevant in the present case: When F_O_ is decoupled from the F_1_-portion, its unitary conductance is approximately 10 fS (for chloroplasts see [30], for *Rhodobacter capsulatus* [31], and for *E. coli* [32]), which implies about 1,000 rounds per second of the c-ring (at 200 mV driving force).

**Figure 1:**
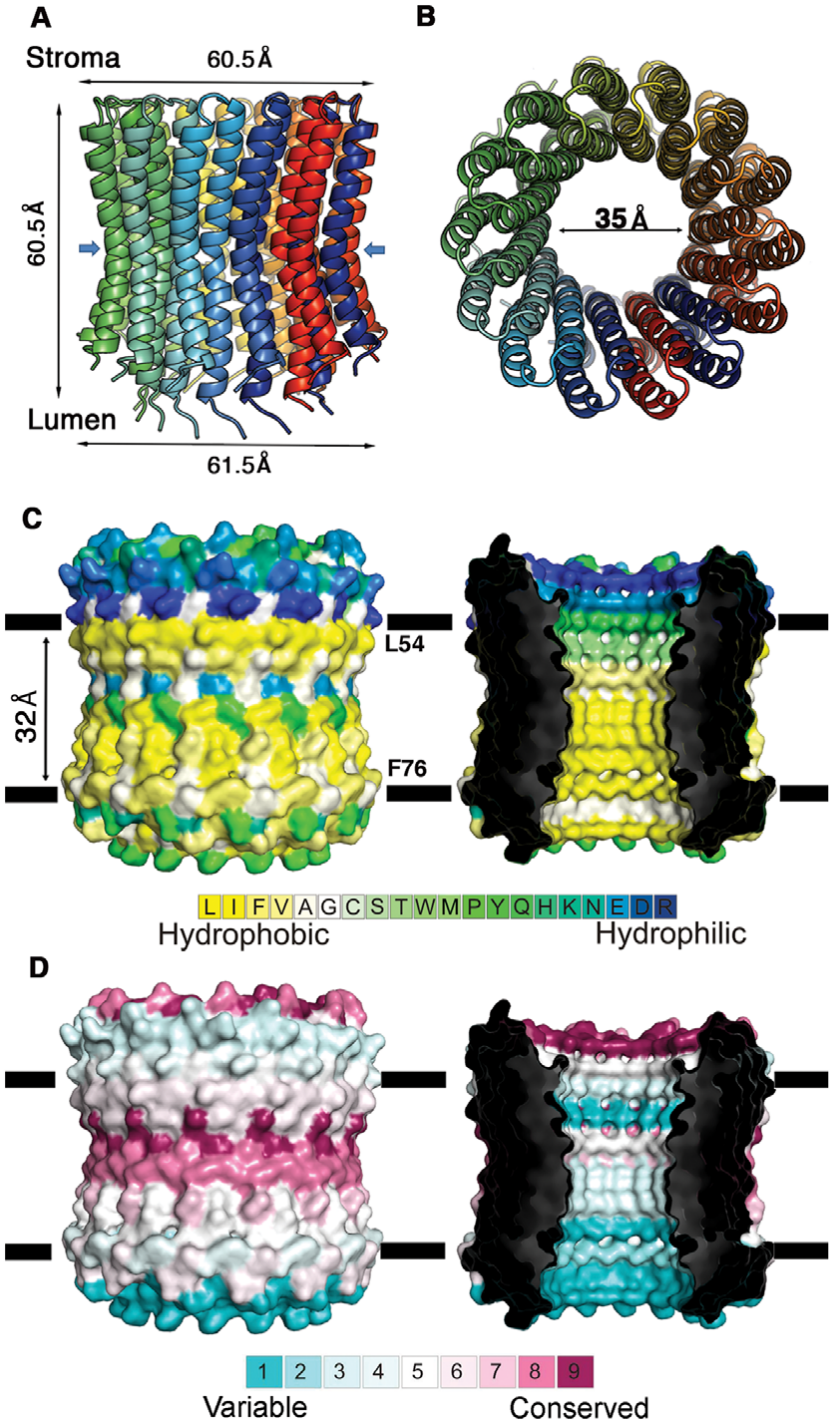
Architecture of the chloroplast ATP synthase subunit-c. **A&B** Side (**A**) and stroma (**B**) views of the ring, with differently colored monomers. The ring dimensions are marked, and blue arrows indicate the narrowest ring region. **C.** Side view of the structure in surface presentation, colored according to the hydrophobicity scale below. Left: the membrane boundaries according to the hydrophobicity profile are marked, mapped to Leu54 and Phe76. The hydrophilic residues reside at the ring edges, corresponding to extra-membrane regions, as well as at the membrane center, at the proton-binding site. Right: slab view, displaying the interior of the ring. **D.** The structure is viewed as in panel C and colored according to evolutionary conservation as calculated by the ConSurf webserver (http://consurf.tau.ac.il, [49]), with cyan-to-maroon indicating variable-to-conserved positions, according to the color bar. Left: the hydrophilic proton-binding site at the membrane center is highly conserved, as are the stroma-facing loops. Right: a slab view reveals that residues lining the interior of the ring are highly variable. Indeed, this region is not expected to possess a functional or structural role.

Simple techniques for coarse-grained simulations of protein dynamics, e.g., elastic network analysis, take just the C_α_-atom of each residue into account, and the solvent is usually neglected. Such an approach does not require extensive computational power and may, in principle, cope with micro- to millisecond events [33]. This restricted approach does not account for the over-damped character of protein dynamics. More advanced techniques have included solvent–protein interactions, damping and thermal properties (for an application to adenylate kinase see [34]); however, simulations are still carried out on a relative and not on a real time scale.

**Figure 2:**
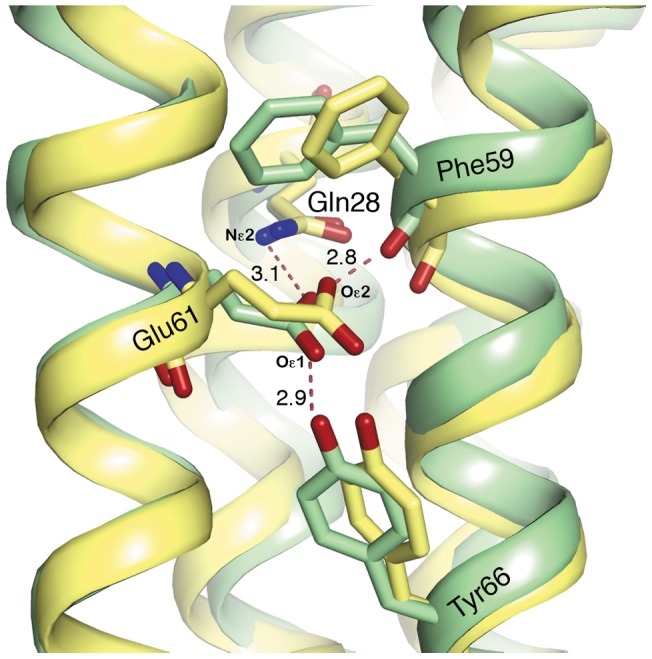
Structural alignment of the c-ring proton-binding site. Structural alignment of the crystal structure of the green pea subunit-c (colored green) and the crystal structure of the protonated state of the cyanobacterium c-ring (PDB ID 2wie, colored yellow). The proton-binding site of the two structures as viewed from the membrane plane is shown, with the lumen below. The residues of the binding site are marked, and the bond length is shown in Å. The alignment indicates that the structural arrangement and chemical coordination of the proton-binding site are conserved between cyanobacteria and higher plants. It should be noted that according to the crystal structure of the spinach c-ring [12], the chemical coordination of the binding site differs from that of the binding sites of the other c-rings shown herein. Yet, MD simulations indicate that chemical interactions similar to those observed for the cyanobacteria and the green pea binding sites are more stable in the spinach site as well [29].

Herein we applied normal mode analysis (without damping) to the c-ring of F_o_. The purpose was to identify the basic deformation modes of the c-ring, and to investigate whether and how the dominant elastic modes might contribute to the function of the ring in this rotary ion-translocator. The method relies on the position of the C_α_-atoms embedded in an elastic network (*in vacuo*) with a single force constant between neighboring nodes. Orthogonal eigen-modes of motion are computed [33]. For various proteins, including membrane channels and transporters, the slowest (i.e., global or cooperative) modes of motion have been shown to represent functionally significant movements [33], [35]. The isotropic Gaussian Network Model (GNM) has been used to assess residue fluctuations and inter-residue dynamical correlations [36], [37], and the Anisotropic Network Model (ANM) has been used to identify motion directionality in three dimensions (3D) [38].

**Figure 3:**
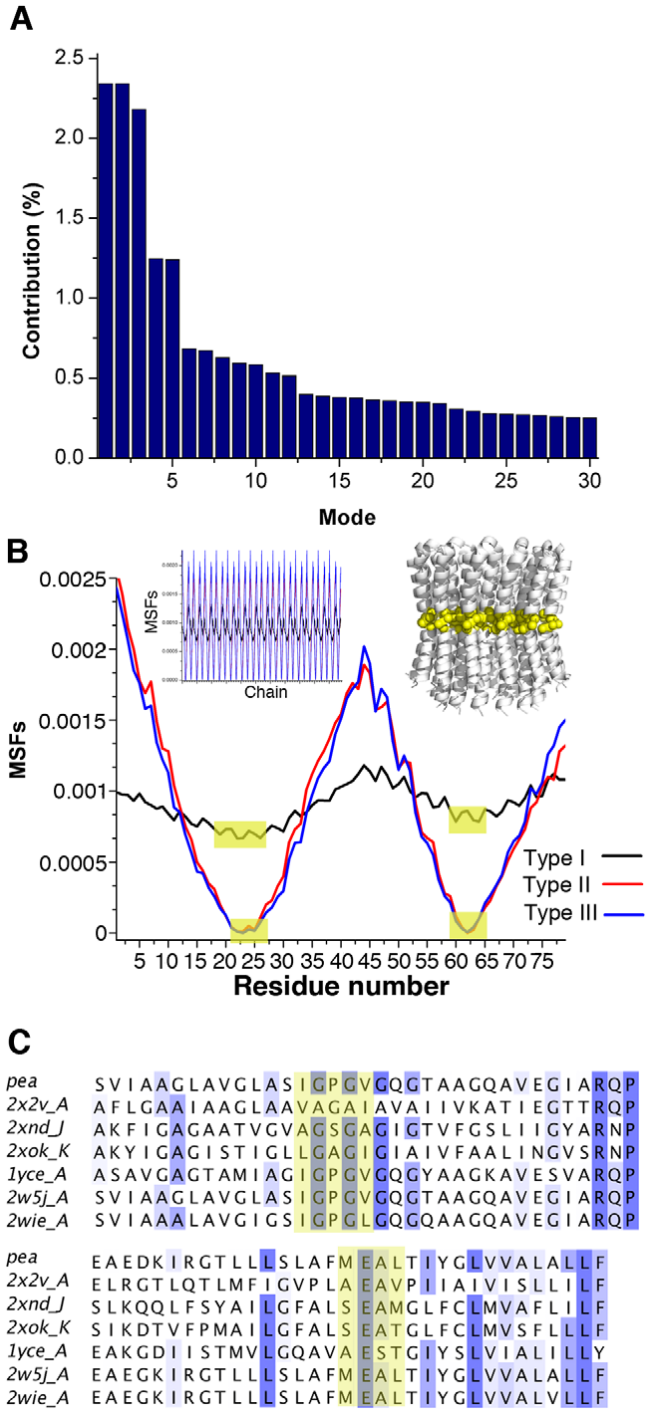
The three classes of motion, derived from GNM: contribution to overall motion, fluctuations and hinges. **A.** Contribution of the 30 slowest GNM modes to the overall motion. The contribution was calculated based on the 1/eigenvalues values of the modes, derived from the GNM calculation. As the mode pairs GNM1-2 and GNM4-5 display the same contribution, i.e. the same eigenvalues, they are considered degenerate modes, with the actual motion consisting of an average of each pair. Overall, there are three main types of motion, denoted as types I, II, III, corresponding to GNM1-2, GNM3 and GNM4–5, respectively. **B.** The mean square fluctuations (MSFs) are shown for one monomer, with the derived hinge regions shaded in yellow. The insert in the upper left shows the MSFs for all monomers, whereas the insert in the upper right displays the c-ring structure from a side view, with the hinge residues as yellow spheres. It is evident that while all three types of motion share the same hinge regions, these are prominent only in motion types II and III and not in type I. **C.** Sequence alignment of c-rings with available structures. The yellow marking indicate the hinge region of types II and III in the GNM analysis of the green pea c-ring. In all rings, the hinge region was assigned to overlapping regions, with the second hinge including the functional glutamate. The alignment displays the region corresponding to positions 9 to 76 of the green pea subunit-c, omitting parts of the N- and C-termini.

We isolated the c-ring of the F_O_F_1_-ATPase of the green pea (*Pisum sativum*) from a native preparation, crystallized it, and determined its homo-tetradecameric structure at a resolution of 3.4Å. The elastic eigen-modes of the ring structure were determined by GNM and ANM, and the same analysis was applied to c-rings from other FoF_1_-ATPases containing 8 to 15 copies of subunit c, respectively, and to a ring of a V-ATPase. We discuss the roles of the dominant eigen-modes in rotary proton translocation, in the stepped torque generation, and in the elastic buffering of the stepped rotation for smooth torque transmission into F_1_.

**Figure 4:**
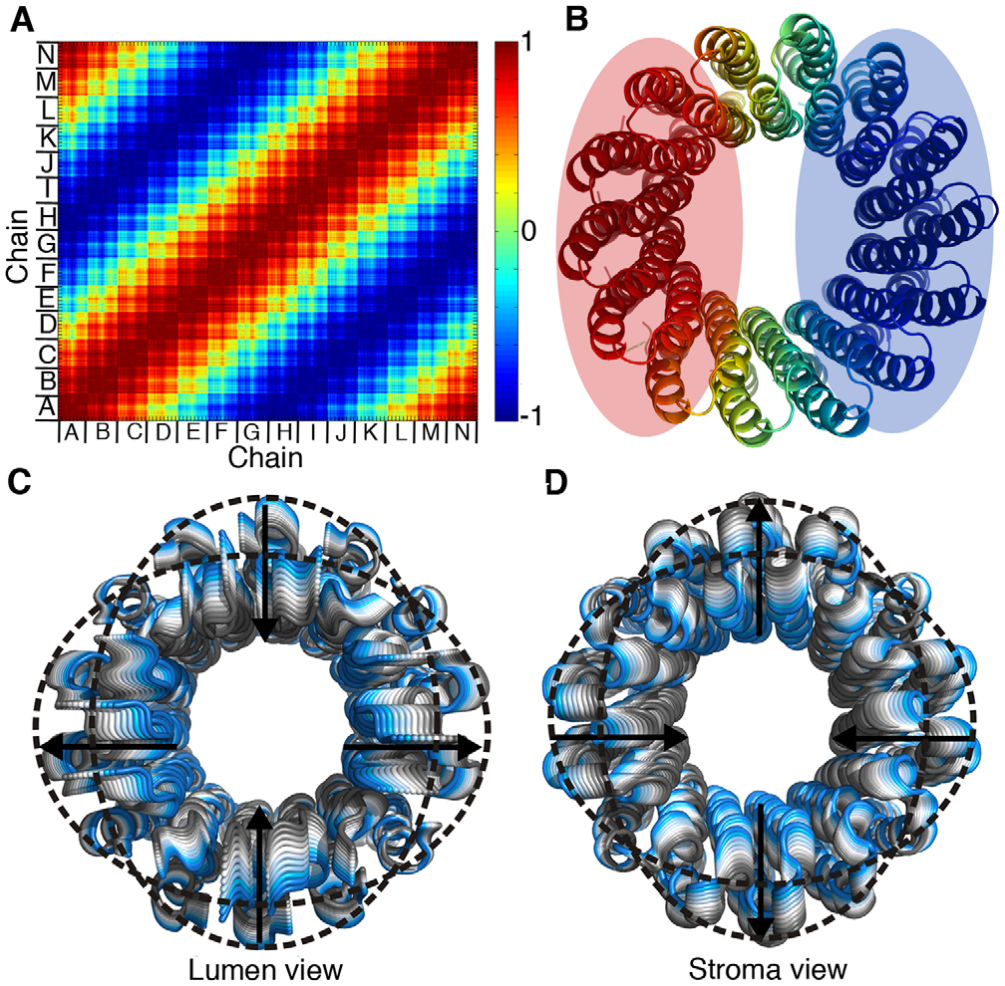
Motion Type I. **A.** Dynamical correlation between all residues in motion type I, derived from GNM. The correlation values range from blue to red, indicating negative and positive dynamical correlation, respectively, according to the scale. The different chains are marked on the matrix. **B.** The GNM dynamical correlation is mapped onto the c-ring. The c-ring is shown in cartoon representation and is viewed from the stroma. **C & D.** The ANM modes corresponding to motion type I. ANM1 (panel C) and ANM3 (panel D) are shown as cartoons and viewed from the lumen and stroma, respectively. The deformations are colored from gray to blue; arrows indicate the direction of motion and dotted circles mark the extreme deformations. In both modes, the c-ring expands and contracts, with oppositely correlated monomers moving towards the ring center, while the rest of the monomers move outwards. This results in an elliptic conformation.

## Materials and Methods

### CF_o_CF_1_ and c-ring purification

Thylakoid membranes were purified from ∼800 g *P. sativum* var. Alaska young leaves as in [39]. CFoCF_1_ complexes were released from the membrane using 0.4% n-dodecyl-β-D-maltoside (DDM, Glycon, Inc.). The preparation was further purified by 2 steps of differential precipitation using PEG 2K as a precipitate using 7% and 9%, respectively. The pellet was dissolved with 20 mM Tricine-Tris (pH 7.4), 0.125 mM dithiothreitol (DTT), and 0.05% DDM, was applied to a 10–40% sucrose gradient containing the same buffer, and was centrifuged using the SW-40 rotor (Beckman Inc.) at 37,000 rpm for 16 h. Fractions containing CFoCF_1_ were pooled together and loaded onto an ion exchange column (DEAE-cellulose, DE52, Whatman, Inc., 1.5×18 cm) pre-equilibrated with 20 mM Tricine-Tris (pH 7.4), 0.125 mM DTT and 0.05% DDM. The column was washed with 40 ml of the same buffer, and fractions containing CF_O_ dominated with subunit-c were eluted by 50–250 mM NaCl linear gradient in 20 mM Tricine-Tris (pH 7.4), 0.125 mM DTT and 0.05% DDM. These fractions were then pooled together and concentrated to 3–4 mg/ml by 2 steps of differential precipitation using PEG 6K as precipitant. The pellet was suspended in 5 mM Tris pH 8.0 and 0.05% Fos-choline 12.

**Figure 5:**
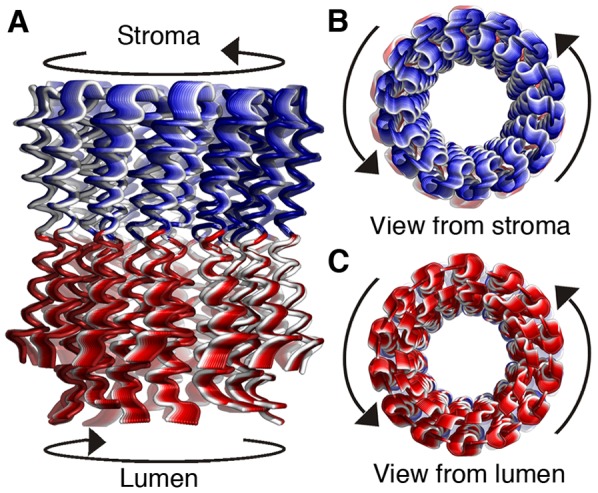
Motion Type II. **A.** The deformations of motion type II (associated with ANM5), colored according to dynamical correlation from GNM. The c-ring is shown in cartoon representation and viewed from the side, with the lumen below. The two dynamical elements identified for this motion-the stroma-facing half and the lumen-facing half, separated by the hinge region at the center of the ring are shaded, respectively, in blue and in red. The deformations, colored from white-to-red and from white-to-blue, show opposite rotational twisting of the two dynamical elements, as marked by the arrows. **B&C** Stroma-facing (**B**) and lumen-facing (**C**) views of the motion as shown in panel **A**, with the arrows indicating the direction of motion.

### Crystallization and structure determination

Subunit-c crystals grew in a crystallization buffer containing 100 mM Na-acetate (pH 4.5), 50 mM MgCl_2_, 50 mM NaCl, 10 mM yttrium chloride, and 14–24% PEG 550 monomethyl ether (MME), reaching their maximal size after 5–7 days. Yttrium was added using a commercial additive kit (Hampton Research) as it improved the shape of the crystals. After the crystals were equilibrated and then incubated for 48 hours at 20°C, the quality of the crystals and the subsequent diffraction pattern were improved by adding to the reservoir 40% PEG 550 MME. The crystals were then flash frozen in liquid nitrogen. Data were collected in ID23-1, ESRF, Grenoble and processed by XDS [40]. Due to anisotropic diffraction, ellipsoid truncation of the data was performed using the UCLA MBI Diffraction Anisotropy Server [41]. Resolution limits were 3.7, 3.6 and 3.4Å along a*, b* and c* axes, respectively. Initial phases were determined by maximum-likelihood molecular replacement as implemented in Phaser [42], [43], using a backbone model derived from the crystal structure of the *S. platensis* c-ring (PDB ID 2wie [24]) as a search model. Iterative refinement (PHENIX [44]) and manual building (Coot [45]) using translation libration screw-motion (TLS) and non-crystallographic symmetry constraints using all fourteen monomers composing the ring produced a model at 3.4Å resolution with R-work/free of 29% and 32%, respectively, with no outliers in the Ramachandran plot. The structure was deposited in the Protein Data Bank under the PDB ID 3V3C.

**Figure 6:**
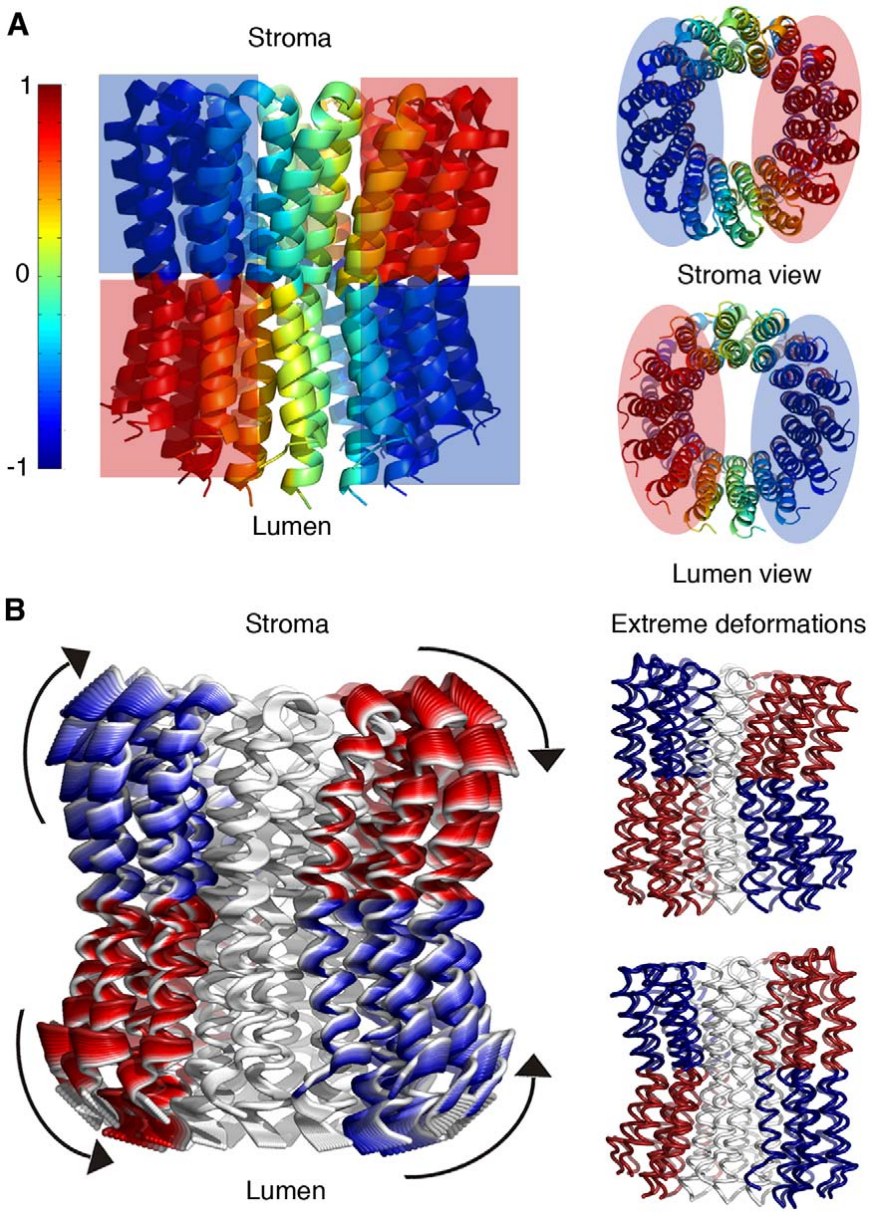
Motion Type III. **A.** The c-ring structure is colored according to the GNM-derived dynamical correlation, with positive-to-negative correlation colored according to the red-to-blue scale. Four main dynamical elements are identified, mapped to the lumen- and stroma-facing halves of the monomers at opposing sides of the ring (marked by red and blue shading). It is apparent that the stroma-facing halves are negatively correlated with their lumen-facing halves, while positively correlated with the lumen-facing halves of monomers situated at the opposing side of the ring. The left-hand side shows a side view, while the right-hand side displays stroma and lumen views. **B.** The deformations of the corresponding ANM motion (ANM6). The structure is colored according to the correlation of the four main dynamical elements identified in panel **A.** Left: ANM deformations, ranging from white to red or from white to blue, with the direction of motion marked. Right: the two extreme deformations, corresponding to the two potential directions of this type of motion, with the main dynamical elements colored red or blue according to their correlation. These deformations describe a bending and stretching motion originating from the hinge at the ring center, with bending of the helices occurring at one side of the ring, while stretching takes place at the opposing side.

### Sequence, evolutionary conservation and pKa calculations

Using the sequence of subunit-c from the green pea, we initiated a BLAST [46] search against the UniRef90 database [47], collecting 351 sequences with Evalue <0.0001. The full sequences were then aligned using MAFFT [48] and input into the ConSurf webserver (http://consurf.tau.ac.il, [49]) to compute conservation scores. The conservation profiles were then mapped onto the c-ring structure.

**Figure 7:**
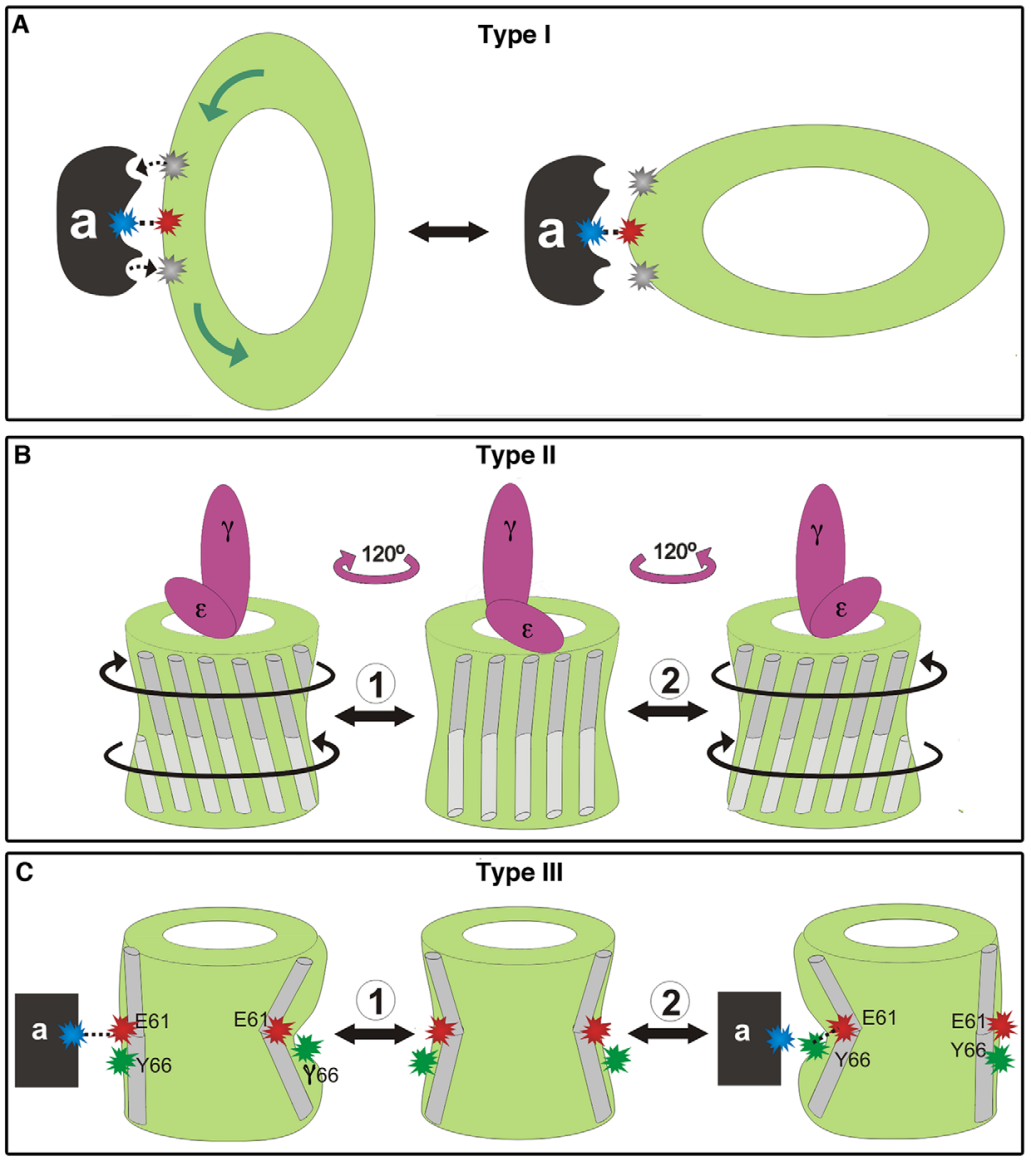
Cartoon illustrating the functional interpretation of the dominant modes. Schematic presentation of the c-ring (light green), subunit-a (dark gray) and the γ-ε complex (pink). Protonated Glu61 of the c-ring, the conserved Arg of subunit-a and Tyr66 are shown as gray, blue and green asterisks, respectively, with unprotonated glutamates as red asterisks. **A. Motion type I.** Top view. Right: Upon ring expansion, the electrostatic interaction between Glu61 and the Arg is strengthened, while the probability of proton transfer is weakened. Left: Upon contraction, c-ring places a flat face toward subunit-a. This interaction could facilitate protonation/deprotonation of Glu61 through the two proton half-channels on subunit-a (dashed black arrows). It results in one step of counter-clockwise rotation (green arrows). The cartoon suggests three sites of interaction between subunits c and a, whereas it has been suggested that the Arg on subunit-a has two functions [65], resulting in two interaction sites. This alternative does not impair the above interpretation regarding effect of this subunit-c deformation. **B. Motion type II.** Side view with the thylakoid lumen below and the stroma side above. Helices are illustrated as gray cylinders, with the lumen-facing halves in lighter shade. As the γ-ε sub-complex undergoes a-120° rotation (marked by pink arrows) and the c-ring rotates by a smaller step [21], the twisting motion of the stroma-facing helical halves (colored dark gray) is suggested to serve as an elastic buffer for smooth torque transfer between F_O_ and F_1_ (marked in directions 1 and 2 by black arrows). The opposite rotational directions illustrate the two potential functionalities, i.e. hydrolysis and synthesis. **C. Motion type III.** Same view as panel B, with TM2 illustrated (light gray). 1. As the structure stretches towards the left side, Glu61 of the leftmost subunit becomes more exposed to the membrane, which could enhance its interaction with the Arg of subunit-a. 2. Bending of the structure masks Glu61 from the membrane, with potential contribution of Tyr66. Again, this motion could facilitate proton exchange between the two residues.

The sequences of the c-rings with available structures were aligned using the 3D-Coffee software package, and the alignment process was further guided by the structural data [50]. Slight manual adjustments were performed in order to achieve better compliance with the known sequence anchors, namely the TM1-TM2 loop and glycine-containing motifs.

pKa values of Glu61 were computed using the PROKA server [51]. Figures were prepared using the PyMol molecular viewer (http://www.pymol.org/).

### GNM and ANM computations

In GNM and ANM, the structure is viewed as a collection of nodes, derived from the C_α_ atoms, and springs, connecting the nodes according to a given distance cutoff [36], [37], [46]. The normal modes of motion are determined by the protein's structural architecture, and are ranked according to their 1/eigenvalues from slow to fast, i.e. from the most cooperative modes to local fluctuations. Based on the modes' contribution to the overall motion, derived from the 1/eigenvalues, we identified five main modes of motion. As observed for other symmetric membrane structures (e.g. [35], [52], [53], [54], [55]), some of the modes were degenerative; two types of motion (types I and III, discussed above) were each derived from a combination of two symmetry-related, GNM modes, exhibiting essentially the same frequency. We thus averaged the GNM fluctuations and inter-residue cross-correlations of each pair of modes to receive symmetrical behavior for all subunits. The three types of motion, referred to as types I, II and III, consist of GNM1-2, GNM3 and GNM4-5, respectively. The hinges were derived from the residue fluctuations of each type of motion, denoted as minima, i.e., regions demonstrating significantly low mobility relative to the rest of the residues. To obtain the directions of these structural displacements in 3D space, we employed ANM using the HingeProt [56] and the ANM webservers [57]. The GNM modes were associated to the ANM modes on the basis of inter-residue cross-correlations (Fig. S4). The same analysis was performed for additional c-ring structures, namely, Protein Data Bank (PDB) IDs 2x2v, 2xnd, 2xok, 1yce, 2w5j and 2wei [12], [23], [24], [25], [26], [58]. Further details are available in the Supporting Text S1.

## Results

### Overall structure of chloroplast ATP synthase subunit-c

The membrane-embedded domain of chloroplast ATP synthase, including subunit-c, was purified from the native holoenzyme by ion exchange chromatography, and further purified in two precipitation steps with polyethylene glycol (See Materials and Methods). This process, in contrast to previous pre-crystallization processes for c-rings [59], [60], was performed without any intense treatment such as harsh detergent or heating. This approach enabled us to crystallize the chloroplast c-ring of a higher plant (*P. sativum*) from its native environment. The crystal structure of the chloroplast ATP synthase c-ring was determined at 3.4Å resolution with R-work/free of 29% and 32%, respectively, with no outliers in the Ramachandran plot (Table 1).

The crystal lattice revealed one tetradecameric ring in the asymmetric unit, forming crystal contacts with three additional rings. One contact region consisted of loop-to-loop interactions mediated by yttrium ions, as indicated by strong electron densities. The remaining interactions involved the N-termini of three monomers with three additional monomers of two symmetry mates. This interaction was mediated by distinct electron density, which was difficult to interpret (Fig. S1A). Interestingly, another crystal contact was generated in the hydrophobic region located between two rings (Fig. S1B). This inter-space was wide enough to incorporate detergent molecules, which can replace the annular layer of thylakoid membrane lipids or the lipids themselves. Indeed, we observed electron density in this region that resembled a lipid-like structure. The onset of the hydrophobic chain was situated parallel to Phe76, mapped to the luminal boundary of the thylakoid membrane, protruding towards the membrane center (Fig. S1B).

The crystal structure of the c-ring from *P. sativum* (see Fig. 1) shows a concave barrel shape with a pronounced waist in the middle, exhibiting tetradecameric symmetry [11] consisting of an inner and an outer ring. The structure was 60.5Å long, with outer ring diameters of 60.5Å and 61.5Å at the stroma and lumen sides, respectively (Fig. 1A), whereas the inner ring had a diameter of 35Å at both sides of the membrane (Fig. 1B). The narrowest diameter, 27Å, was mapped to the middle of the ring at Ile22 and Gly23 (Fig. 1A). Each monomer was a hairpin-shaped, 81-residue polypeptide, containing two trans-membrane (TM) helices; TM1 (residues 4–41) composed the inner ring, and TM2 (residues 46–75) formed the outer ring. Both the C- and N-termini were positioned towards the lumen. TM1 and TM2 were connected by a short hydrophilic loop located at the stroma (residues 42–45). TM1 and TM2 were both kinked towards the membrane (with angles of 31° and 20°, respectively), with the kink mapped to the region around Gly23 and Glu61.

According to the hydrophobicity profile [61], residues Leu54 and Phe76 line the hydrophobic boundaries of the c-ring, surrounded by hydrophilic residues on both ends (Fig. 1C). This indicates that the core membranal region of the c-ring spans 32Å, which potentially correspond to the hydrophobic core width of thylakoid membranes at the ATP synthase location. Additionally, a pronounced hydrophilic patch is apparent at the center of the membrane region, representing the proton-binding site, which is further described below (Fig. 1C). Evolutionary conservation analysis, mapped onto the c-ring structure, was in agreement with the observed structural and functional features. The highly conserved residues (receiving ConSurf grades of 8 or 9 [49]) included the hydrophilic membrane-exposed binding site and surrounding residues, as well as the stroma-facing loop (Fig. 1D). The latter is implicated in the interaction with the γ-ε globular region, part of the F_1_ component [6]. Additional conserved positions were detected at helix-helix interactions. Variable positions, i.e., those receiving ConSurf grades of 1 or 2, mapped to the remaining lipid-exposed residues (Fig. 1D), as well as to positions facing the ring interior, a region that probably does not possess a functional or structural role (Fig. 1D).

The proton-binding site retains a chemical coordination similar to that of the alkalophilic cyanobacterium *Spirulina platensis* (Fig. 2), including the same amino acids: Gln28 and Glu61, located on TM1 and TM2, respectively, of the same monomer, and Phe59 and Tyr66, which are positioned on TM2 of an adjacent monomer. All four residues were clearly identified in the electron density map (Fig. S2). A network of hydrogen bonds stabilizes the highly conserved Glu61 (Fig. 2).

Glu61 is likely to be protonated in the structure, which was crystallized at pH 4.5, and this notion is supported by a pKa analysis [51]. The Oε2 part of the carboxyl group of Glu61 generates hydrogen bonds with the Nε2 of Gln28 and the carbonyl group of Phe59 (with distances between relevant atoms of 3.1Å and 2.8Å, respectively). The second oxygen group, Oε1, forms a hydrogen bond with the hydroxyl group of Tyr66 (2.9Å). This chemical coordination represents a locked conformation of the binding site [24].

### Normal mode analysis

We analyzed the cooperative movement of the c-ring structure in terms of elastic network models. The motion patterns of residues and the cross-correlations between residues were computed using GNM [36], [37]. The directionality of this motion was derived from associated ANM modes [38]. We focused on the five dominant modes, which fell into three classes, referred to as types I, II and III (Fig. 3A). Type I was two-fold degenerate, and the associated deformation of the ring ellipsoidal. Type II was undegenerate and the deformation torsional, and type III was two-fold degenerate with kinks in the middle.

We carried out the same normal mode analysis for the structures of six other c-rings of FoF_1_-ATPases, in which the respective numbers of c-subunits varied from eight to fifteen, as well as for the functionally-related ring of a V-ATPase, comprising monomers of four TM helices each [27]. All revealed very similar modes to those of the green pea c-ring, with the same three types of motion (see Fig. S3, Table S1). The similarity of the dominant slow modes among these different enzymes was not very surprising considering their common toroidal topology. Comparable modes have been reported for an unrelated toroid, the nuclear pore complex [62].

In all three types of motion of the *P. sativum* c-ring, the hinge regions, i.e. the least mobile residues in the GNM analysis, were in similar locations. They were clustered at positions 21–26 and 60–64, approximately at the kinked central regions of the two TM helices (Fig. 3B and 3C). Notably, the essential acidic residue of the proton-binding site, Glu61, was part of the hinge region. Gln28 and Phe59, also involved in the ion-binding site [24], resided in close proximity to the hinge. The hinge region was more pronounced, i.e. less mobile, in motion-types II and III, and was less pronounced in type I (Fig. 3B). Although all three types of motion shared the same hinge region, the inter-residue correlations significantly differed (Figs. 4A, 4B, 5, 6A and S4). Typically, GNM and ANM modes are matched according to the residue fluctuations, specifically, the location of the hinges. In this analysis, however, as the hinge locations were essentially the same in all classes of motion, they could not be utilized to correlate the GNM and ANM results. We thus relied on the inter-residue correlations to associate ANM modes to each type of GNM-derived motion (Fig. S4).

### Type I

In this type of motion, the inter-residue correlation separated the c-ring into two oppositely-correlated dynamical elements, divided by a plane perpendicular to the membrane (Figs. 4A and 4B). The associated ANM modes (ANM1–4; Fig. S4 and Table S1) manifested an ellipsoidal deformation, emphasized at the lumen-facing ends in ANM1 and ANM2, and at the stroma-facing loops and helical regions in ANM3 and ANM4 (Figs. 4C, 4D and Movies S1 and S2). In this motion, the ring expands and contracts; opposing monomers approach the center of the c-ring, while the rest of the monomers simultaneously move outwards. This motion changes the overall shape of the ring, transforming its initial round shape into an elliptic one.

### Type II

In this motion, the c-ring structure was divided into two dynamical elements, with a plane separating the helices into lumen- and stroma- facing halves, passing through the hinge region at the membrane center (Figs. 3B and 5A). One dynamical element consisted of the helices' lumen-facing halves (residues 3–21 and 63–81), oppositely correlated to the second element, which includes the stroma-facing halves and the short loop (residues 24–60) (Fig. 5). Correspondingly, the matched ANM mode (ANM5; Fig. S4 and Table S1) displayed a rotational motion of the two dynamical elements in opposite directions (Fig. 5 and Movie S3), referred to as a twisting motion. The lumen-facing halves rotated clockwise while the stroma-facing halves simultaneously rotated counter clockwise, and *vice versa*.

### Type III

The inter-residue correlation divided the structure into four main dynamical elements. Each of these four elements consisted of either the lumen- or the stroma-facing halves of approximately five monomers, divided by the hinges at the middle plane of the membrane (Figs. 3B and 6A). Negative dynamical correlation was detected between stroma- and lumen-facing halves of the same five monomers (Fig. 6A). Positive dynamic correlation was observed between stroma-facing halves and lumen-facing halves situated on monomers of opposing sides of the ring (Fig. 6A). The matched ANM modes (ANM6–9; Fig. S4 and Table S1) consisted of a bending and stretching motion, governed by the hinges at the center of the ring (Figs. 3B and 6B, Movie S4). The four dynamical elements described above endured the largest structural displacements during the motion, while the monomers between them mediated the motion. This bending and stretching motion altered the exposure of the hinge region at TM2 towards the membrane.

## Discussion

This work presents a hybrid approach of X-ray crystallography and computational analysis to reveal both fine and gross structural properties of the c-ring of the FoF_1_-ATPase in higher plant chloroplasts. We discuss the three types of elastic slow modes and associate them with the rotary ion translocating function of the c-ring, in interaction with subunits a and bb'.

### Crystal structure of the c_14_-ring of the F_O_F_1_-ATPase in the pea

High-resolution crystal structures of the FoF_1_-ATPase c-rings of various organisms have been determined [6], [12], [23], [24], [25], [26]. The ring's resilience to high temperature and harsh detergents has facilitated the preparation of 2D-crystals for atomic force microscopy and electron microscopy and of 3D-crystals for X-ray structure analysis. It is an interesting possibility that crystallization under harsh conditions “purifies” certain structural variants. To address this, we used a very mild preparation and crystallization technique, starting from the native holoenzyme. In order to overcome the common heterogeneity of plant material, *P. sativum* var. Alaska was used under controlled growth conditions. By utilizing a mild approach, we hoped to capture a native form of the c-ring structure, revealing the native protein/lipid-interaction. To the best of our knowledge, the special electron density detected at the crystal contacts between hydrophobic regions of the c-ring is a unique feature, which has not been observed to date in other crystal structures of membrane proteins (Fig. S1B). A galactolipid was tentatively modeled into the intercalating electron density, in compliance with the notion that the uncharged heads of the galactolipids enable their penetration through the thylakoid membrane, as was demonstrated in the crystal structure of cyanobacterial photosystem II [63]. We expect that our preparation and crystallization approach would be useful for future investigations aimed at exploring the effect of lipids on the functionality of membrane proteins. Moreover, it could be highly effective for studying and determining the structure of the a-c complex of the FoF_1_-ATPase.

Despite the high sequence identity between the c subunit in the green pea and the c subunits of other photosynthetic organisms (e.g., subunit-c of spinach spp. [12] and *S. platensis* [24], both displaying more than 85% identity to the green pea subunit-c), there are distinguishable structural differences among them. The proportions of the *P. sativum* c-ring (height/width 60.5Å/60.5Å) differ slightly from those of its spinach homologue (65Å/58Å, respectively [12]), while its cyanobacterial homologue, with 15 monomers on the ring, is similar (65Å/65Å, [24]). Similarly, the coordination pattern at the proton-binding site in the pea c-ring resembles that in the cyanobacterium (Fig. 2) yet differs from that of the spinach homologue. In the latter structure, additional hydrogen bonding was detected between the hydroxyl groups of Thr64 with Oε1 of Glu6, and Gln28 was not detected in the electron density map [12]. Recent MD simulations indicate, however, that the original coordination of the proton-binding site is unstable, and therefore rapidly incorporates the bacterial-like coordinates [29]. In sum, the F_o_F_1_-ATPase c subunits derived from the chloroplasts of higher plants and from their ancestors, the oxygenic photosynthetic bacteria, share a chemical coordination and a hydrogen bond network that stabilize proton binding by the essential glutamate on subunit c.

### Network model to assess the dominant elastic modes of the c-ring

The elastic network computational approach we used was based on the coarse-grained topology of the protein. It accounts only for the C_α_-atoms and yields the eigen-modes of an undamped elastic network. In several cases it has been demonstrated that the few dominant (slow) eigen-modes observed for a given protein can be associated with large-scale and long-range functional movements that are pivotal for that protein's mechanism [33], [35]. In a real protein that is embedded in a solvent (the viscous membrane in this case), the elastic vibrations of each mode would be overdamped. Instead of oscillating, the domains stochastically fluctuate in the elastic potential well associated with a given mode.

The normal mode analysis in the present work was restricted to the isolated c-ring of the green pea chloroplast. The c-ring's interactions with both subunits a and bb' in the membrane, and with γ and ε of the F_1_-portion, were neglected. We assumed that these intrinsic classes of motion of the c-ring are maintained within the entire c_14_abb'-complex. This assumption is supported by the literature, which has shown that isolated units intrinsically display the motion corresponding to that of the physiologically-relevant complex. GNM fluctuations of isolated monomers and those derived from a complete complex have been successfully employed to assess docking models [30].

We applied the same network analysis to c-ring structures from other organisms, varying in size, shape and the number of monomers in the c-ring (Figs. 3C and S4, Table S1). These analyses yielded the same patterns of slowest modes. Similar results have been obtained for other, unrelated toroid structures, for instance the nuclear pore complex [62].

### Functional interpretation of the three types of motion

The mechanism of rotary proton translocation and torque generation relies on the interaction between the ring of c-subunits and subunits a and bb'. The interplay between firm attachment and rotary fluctuations of the c-ring and subunits a and bb' has remained to be characterized in detail. It has not yet been determined whether this interplay is mainly governed by (i) local conformational fluctuations (e.g., of residues in the ion-binding pocket of subunit c [64], or of the five helices of subunit a), or (ii) more global movements of the c-ring, as emphasized in this work. In the following, we tentatively correlate the three classes of dominant global modes of the c-ring with its rotary function in F_O_F_1_ and suggest a possible functional role.

### Motion of type I

The contraction and expansion of the ring deform the ring elliptically (Figs. 4C and 4D, Movies S1 and S2). Expansion of the ring towards the a-subunit could favor closer apposition of the ion binding pocket on subunit-c towards the essential arginine on subunit-a. This might strengthen the electrostatic interactions between the unprotonated Glu61 and the arginine (Fig. 7A, right). On the other hand, the apposition of the flat face of the ring might facilitate the contact of Glu61 for protonation/deprotonation through either of the proton half-channels (Fig. 7A. left). The deprotonation of one Glu through one half-channel together with the protonation of the previously deprotonated Glu on the adjacent monomer on the ring imply changing the c-subunit copy that interacts electrostatically with the essential arginine on subunit a. In other words, this motion advances the rotation of the c-ring by one step.

### Motion of type II

Motion of type II twists the stroma- and the lumen-facing surfaces against each other around the ring's axis (Figs. 5 and 7B, Movie S3). It is conceivable that this type of deformation contributes to the previously established elastic buffer between F_o_ and F_1_ [17], [19], [20], [21]. The latter studies do not establish the extent to which subunits γ or ε of F_1_ or the c-ring contributes to the high elastic compliance of this buffer. Based on the present analysis, we propose that the twisting deformation of the c-ring (Fig. 5) is part of the elastic buffer between ATP synthesis and proton transport (Fig. 7B).

### Motion of type III

Motion of type III bends and stretches the structure as displayed in Figures 6B and 7C (see Movie S4). This motion is expected to affect the exposure of Glu61, situated at the hinge point. Upon proton release and before binding to subunit-a, negatively charged Glu61 could possibly become exposed to the membrane environment, which is thermodynamically unfavorable. The bending of the helices around the hinge position (Fig. 6B and Movie S4) could thus partially shield the charged acidic residue from the hydrophobic lipids, stabilizing this intermediate state. We suggest that Ala58, Ile65 and Tyr66 might play key roles in this process, as these are highly conserved residues around Glu61 according to our conservation analysis (Fig. 1C). Specifically, hydrophilic Tyr66 might change its original interaction with Glu61 (Fig. 2), masking it from the hydrophobic environment via its bulky ring (Fig. 7C). Interestingly, when simulating a deprotonated state of the glutamate, Pogoryelov et al. observed that the helix carrying the deprotonated glutamate is more strongly kinked [64], which is compatible with the present analysis. Our results, however, cannot account for straightening of an adjacent helix encompassing a protonated glutamate, also demonstrated by the above researchers' simulations. The stretching of the helices, on the other hand, could increase the exposure of Glu61 towards the membrane. This, in turn, could position the deprotonated Glu61 closer to the essential positive charge on subunit-a (Figs. 6B and 7C).

## Conclusion

Elastic network analysis of the c*_n_*-ring of the FoF_1_-ATPase demonstrated five dominant modes of motion, and we interpreted the roles of these modes in the interaction of the c-ring with its counterpart in Fo (the a and bb' subunits) and with subunits γ and ε of F_1_. Thus, these modes affect (i) the dynamics of sliding versus binding of c*_n_* relative to abb', (ii) proton transfer between c and a, and across the membrane, and (iii) the elastic torque transmission between Fo and F_1_. The coarse-grained elastic network formalism yields global modes of elastic vibrations. For a protein that is embedded in a solvent (here the membrane), these vibrations are overdamped, and the harmonic dynamics are converted into stochastic fluctuations. We suppose that the relaxation times are shorter than the transit time of proton transfer, which is approximately 10^−4^s (at 200 mV driving force) [31]. Although the conformational fluctuations may facilitate rotary proton transfer and torque generation by Fo, they do not limit the rate, as evident from the ohmic character of proton conduction by Fo [31].

## Supporting Information

Figure S1 **Crystal contacts and packing.** The c-ring is viewed in cartoon representation. **A.** Crystal contacts between the c-ring (blue) and its adjacent symmetry mates (green), with interactions of either the stroma loops or the N- and C- termini. **B.** Crystal contacts at the hydrophobic region between adjacent rings, mediated by density corresponding to single lipid molecule. A di-galactolipid is modeled within the lipid density. The hydrophobic moiety of the lipid is situated exactly at the predicted hydrophobic core of the membrane (Fig. 1C). Simulated annealing omit map (Fo–Fc) contoured at 1σ.
(TIF)

Figure S2 **Electron density map of the proton binding site.** Side view of the c-ring, with the lumen below. The binding site residues are shown as sticks, with their attributed electron density shown as mesh. Density map (2Fo–Fc), contoured at 1.3σ.
(TIF)

Figure S3 **Comparison of the slowest types of motion of the green pea c-ring, c-ring of** ***Bacillus pseudofirmus*** **(PDB ID 2×2v) and c-ring of the bovine F1-c8 sub-complex (PDB ID 2xnd).** The deformations of the corresponding types of motion (Table S1) are shown, colored by their GNM-derived cross-correlations, according to the color bar below, with negative (blue) to positive (red) correlation ranges between -1 and 1. Arrows indicate the direction of motion. This comparison shows that although the rings are of different sizes and shapes, their three slowest types of motion correspond to each other. Similar results were obtained for the rest of the rings (Table S1). Note that for PDB ID 2xnd, the order of modes differs from that of the green pea c-ring, although the types of motion are the same (Table S1).
(TIF)

Figure S4 **Association of GNM and ANM modes.** As all types of motion consisted of the same hinge regions (Fig. 3A), we matched the GNM and ANM modes using their inter-residue cross-correlations. For motion type I, derived from the average GNM1-2 mode, ANM1 displayed a very similar cross-correlation distribution. The exact same matrix was observed for ANM2, ANM3 and ANM4 (data not shown), indicating that these modes correspond to GNM1-2 as well. The inter-residue correlation of GNM3 (motion type II) was matched to ANM5, whereas GNM4-5, representing motion type III, was associated with ANM6, as well ANM7, ANM8 and ANM9, manifesting the same motion.
(TIF)

Text S1 **Normal mode analysis: GNM and ANM.**
(DOCX)

Table S1 **Comparison between normal mode analyses of different c-rings.** For each ring [9], [10], [11], [12], [13], [14], [15], the GNM and ANM modes corresponding to motions of types I, II and III are specified (colored green, blue and red, respectively). Modes were matched according to the ANM deformations as well as the GNM cross-correlations, as exemplified in Figure S4.
(DOCX)

Movie S1 **Motion type I – ANM1.** The deformations of ANM1, matched to the first type of motion, are shown as cartoons and viewed from the lumen. The coloring corresponds to the inter-residue correlation of chain A of GNM1-2, ranging from red to blue (Figs. 4A and 4B). The motion manifests extraction/contraction of the ring emphasized at the lumen end, with oppositely correlated dynamical elements from the two sides of the ring simultaneously approach the ring center and move away from it.
(MOV)

Movie S2 **Motion type I – ANM3.** Same as Movie S1, with ANM3 viewed from the stroma. This shows the extraction/contraction motion manifested at the stroma-facing ends.
(MOV)

Movie S3 **Motion type II – ANM5.** The ANM5 deformations are viewed as cartoons from the side, with the lumen below. The colors, corresponding to GNM3 (Fig. 5), depict two oppositely correlated dynamical elements, consisting of the lumen- and stroma-facing halves, separated by the hinge at the ring center. The two dynamical elements undergo twisting motions in opposite directions.
(MOV)

Movie S4 **Motion type III – ANM7.** The deformations predicted by ANM7 are shown from the side and colored by the cross-correlations of the N-terminal of chain A with the rest of the structure, blue-to-red indicating negative-to-positive correlation. The motion consists of bending and stretching of the structure towards the membrane, affecting the exposure of the hinge region, including Glu61.
(MOV)
