# Supplementary figures and images for: Structure and Flexibility of the C-Ring in the Electromotor of Rotary FoF1-ATPase of Pea Chloroplasts
Source: PLoS One. 2012 Sep 25;7(9):e43045. doi: 10.1371/journal.pone.0043045 (PMC3458034; doi:10.1371/journal.pone.0043045)

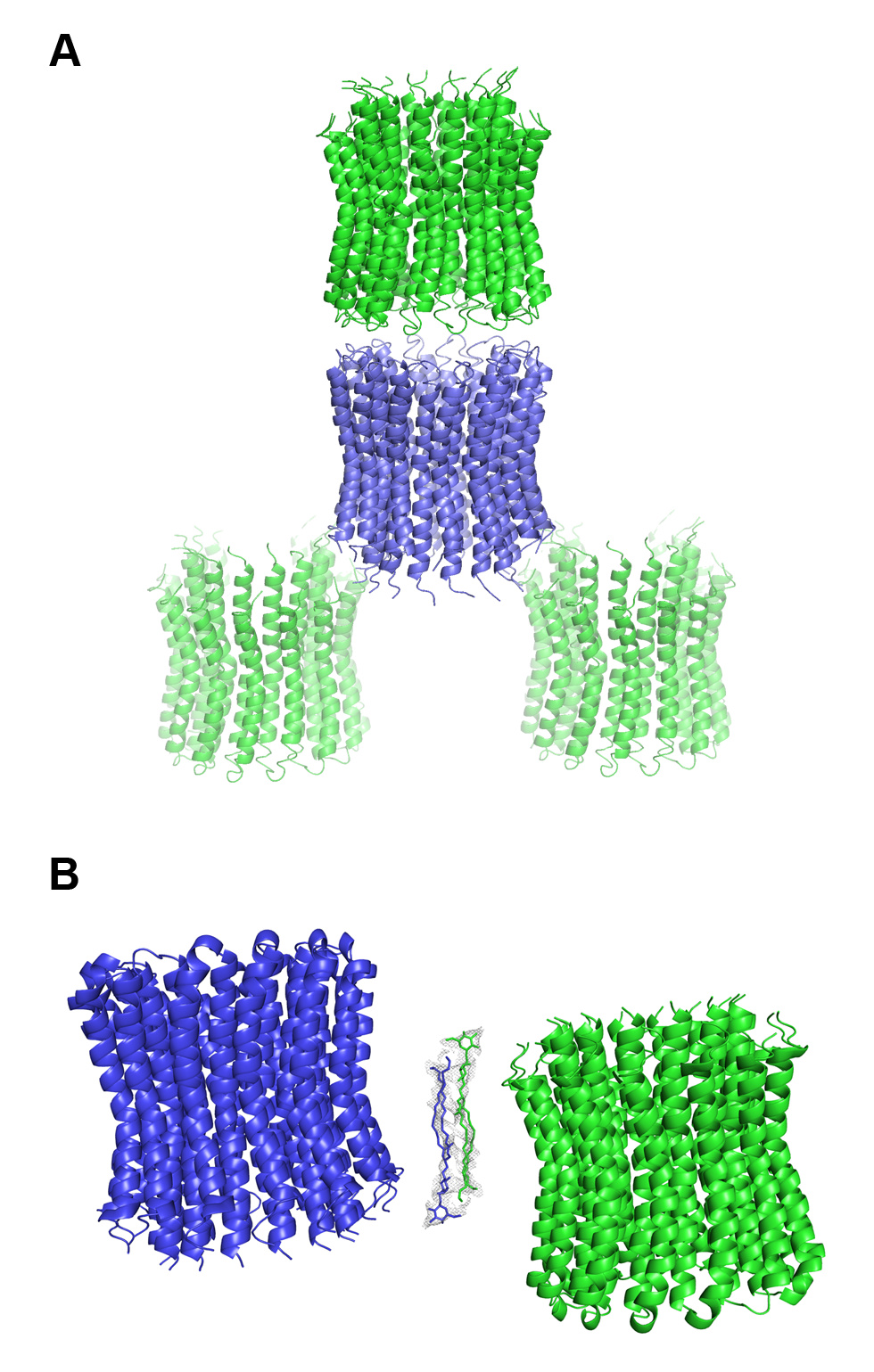

Supplement: Figure S1 — Crystal contacts and packing. The c-ring is viewed in cartoon representation. A. Crystal contacts between the c-ring (blue) and its adjacent symmetry mates (green), with interactions of either the stroma loops or the N- and C- termini. B. Crystal contacts at the hydrophobic region between adjacent rings, mediated by density corresponding to single lipid molecule. A di-galactolipid is modeled within the lipid density. The hydrophobic moiety of the lipid is situated exactly at the predicted hydrophobic core of the membrane (Fig. 1C). Simulated annealing omit map (Fo–Fc) contoured at 1σ. (TIF) [file pone.0043045.s001.tif]

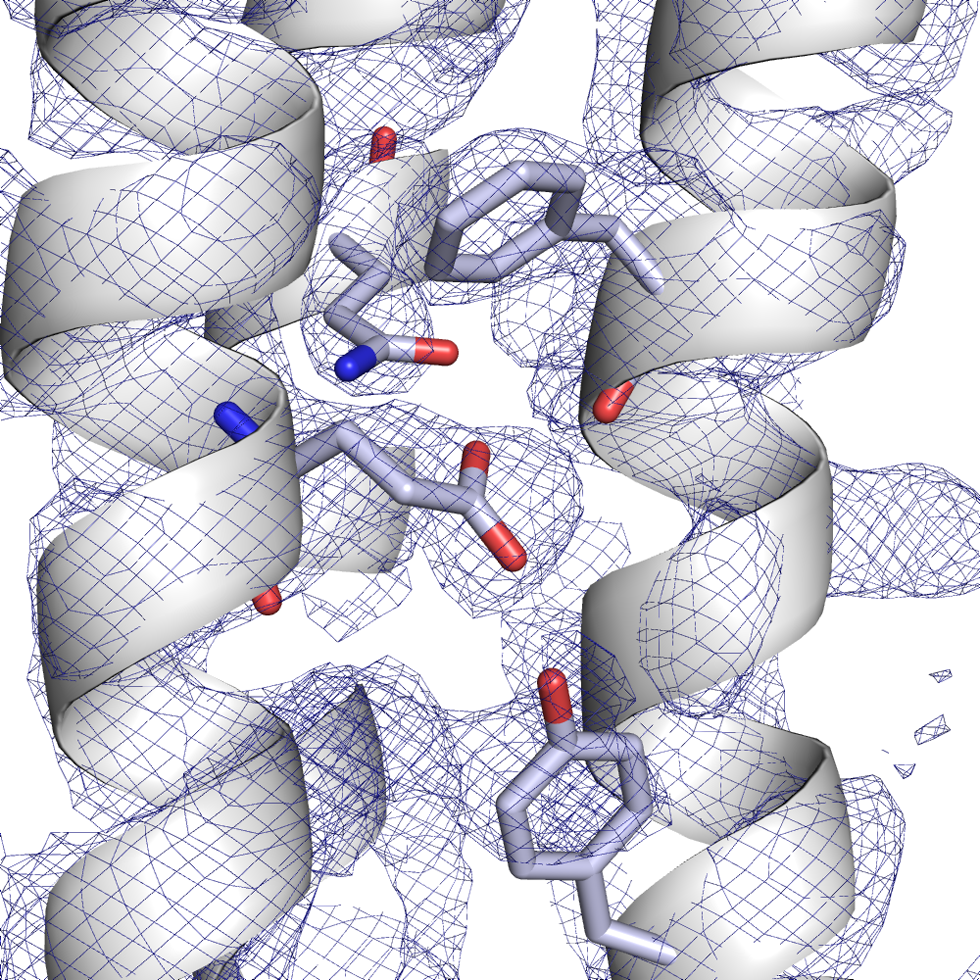

Supplement: Figure S2 — Electron density map of the proton binding site. Side view of the c-ring, with the lumen below. The binding site residues are shown as sticks, with their attributed electron density shown as mesh. Density map (2Fo–Fc), contoured at 1.3σ. (TIF) [file pone.0043045.s002.tif]

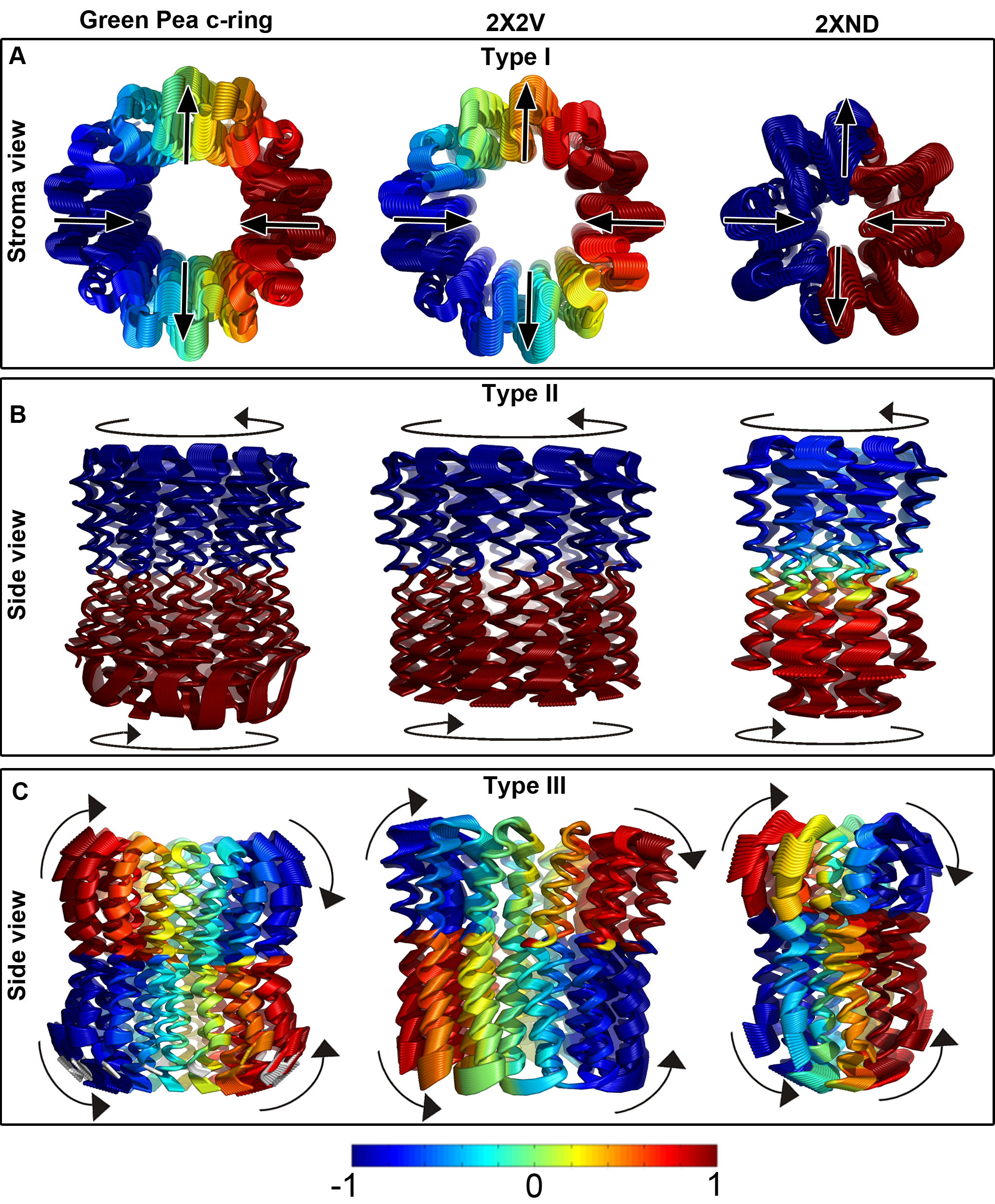

Supplement: Figure S3 — Comparison of the slowest types of motion of the green pea c-ring, c-ring of Bacillus pseudofirmus (PDB ID 2×2v) and c-ring of the bovine F1-c8 sub-complex (PDB ID 2xnd). The deformations of the corresponding types of motion (Table S1) are shown, colored by their GNM-derived cross-correlations, according to the color bar below, with negative (blue) to positive (red) correlation ranges between -1 and 1. Arrows indicate the direction of motion. This comparison shows that although the rings are of different sizes and shapes, their three slowest types of motion correspond to each other. Similar results were obtained for the rest of the rings (Table S1). Note that for PDB ID 2xnd, the order of modes differs from that of the green pea c-ring, although the types of motion are the same (Table S1). (TIF) [file pone.0043045.s003.tif]

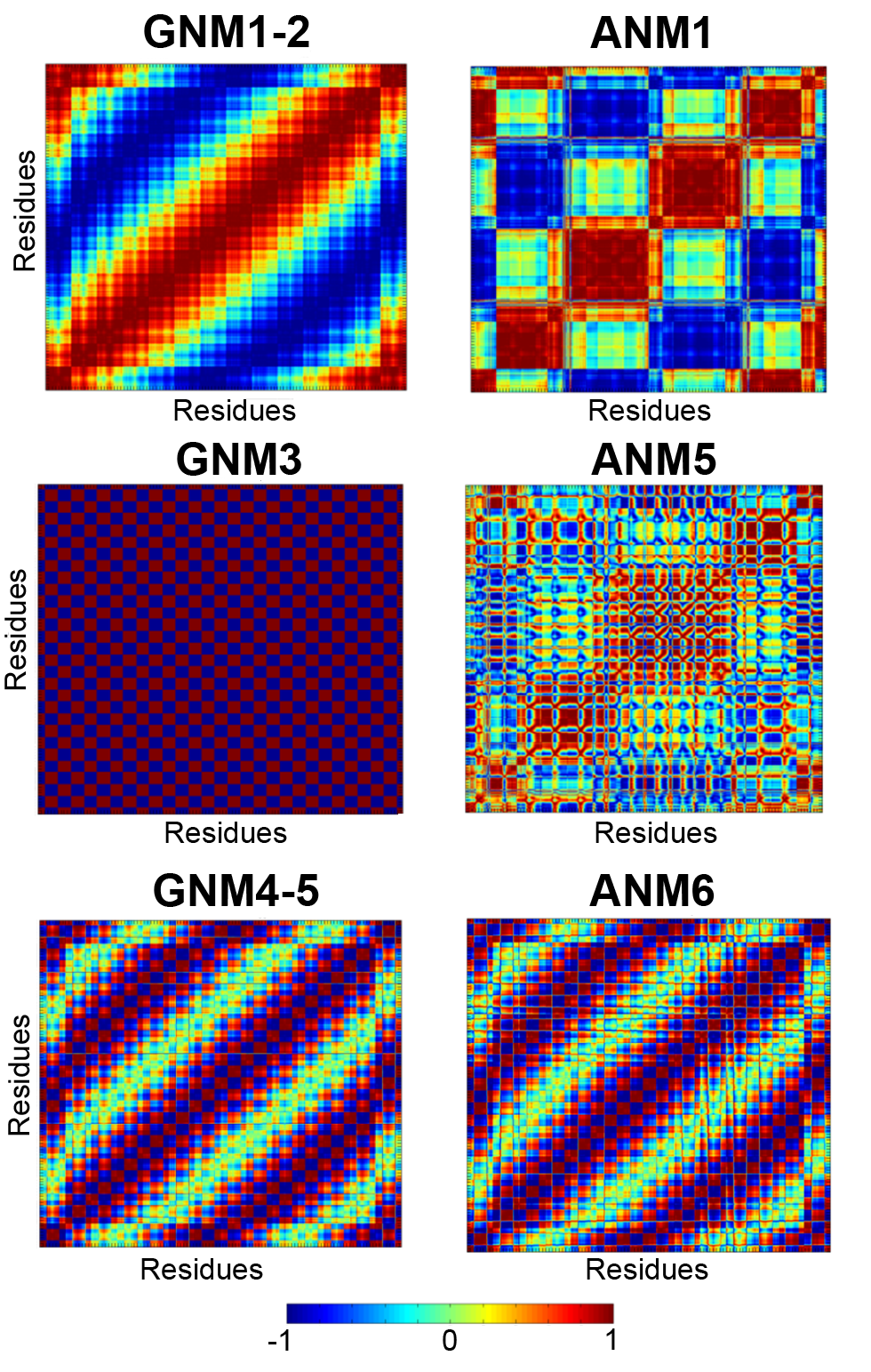

Supplement: Figure S4 — Association of GNM and ANM modes. As all types of motion consisted of the same hinge regions (Fig. 3A), we matched the GNM and ANM modes using their inter-residue cross-correlations. For motion type I, derived from the average GNM1-2 mode, ANM1 displayed a very similar cross-correlation distribution. The exact same matrix was observed for ANM2, ANM3 and ANM4 (data not shown), indicating that these modes correspond to GNM1-2 as well. The inter-residue correlation of GNM3 (motion type II) was matched to ANM5, whereas GNM4-5, representing motion type III, was associated with ANM6, as well ANM7, ANM8 and ANM9, manifesting the same motion. (TIF) [file pone.0043045.s004.tif]
