# Supplementary material for: Structure and Flexibility of the C-Ring in the Electromotor of Rotary FoF1-ATPase of Pea Chloroplasts
Source: PLoS One. 2012 Sep 25;7(9):e43045. doi: 10.1371/journal.pone.0043045 (PMC3458034; doi:10.1371/journal.pone.0043045)
Supplement: Text S1 — Normal mode analysis: GNM and ANM. (DOCX) [file pone.0043045.s005.docx]

**Supporting text S1**

**GNM versus ANM**

In GNM and ANM [[1](#_ENREF_1),[2](#_ENREF_2),[3](#_ENREF_3),[4](#_ENREF_4)] the Cα atoms of the structure are viewed as a collection of nodes, with springs connecting the Cα-atoms within a specified distance range. The distinct architecture of the structure defines its collective modes of motion. As GNM is an isotropic and one-dimensional model, it is only capable of characterizing the fluctuations’ magnitudes, while ANM can also predict the directions of the fluctuations. In GNM the correlation between the fluctuations Δ**R**_i_ and Δ**R**_j_ of residues i and j are expressed as the sum of individual modes, where N is the number of residues, using:

< Δ**R**_i,_ Δ**R**_j_> = (3k_B_T/γ) [**Γ**^-1^]_ij_ = (3k_B_T/γ) Σ_k_  [λ_k_^-1^ **u**_k_ **u**^T^_k_ ]_ij_ (1)

where **Γ** is the connectivity (or Kirchhoff) matrix, representing the residues’ interaction within a distance cut-off using a harmonic potential function with a force constant γ. The term **k** denotes the N-1 nonzero modes; λ**_k_** is the **Γ** eigenvalue referring to the k-th mode of motion; **u**_k_ is the k-th eigenvector; k_B_ is the Boltzmann constant; and T is the absolute temperature. The contribution of each mode to the overall motion is scaled according to the inverse value of the mode’s frequency, i.e. its eigenvalue. For symmetric structures, symmetrically degenerate modes can often be identified, recognized as two or more modes with the same eigenvalue (or contribution) that demonstrate dissimilar fluctuations for symmetric subunits. When averaging these modes, we can obtain the desired symmetric behavior. When i=j the computed correlations correspond to each residue’s mean-square fluctuation. For inter-residue cross-correlations, normalized positive and negative correlation values reflect residue fluctuations correlated in the same and opposite directions, respectively, computed according to the following equation:

< Δ**R**_i_ Δ**R**_j_>_norm_ = <Δ**R**_i_ Δ**R**_j_>/(<Δ**R**_i_ ^2^>^1/2^<Δ**R**_j_^2^>^1/2^) (2)

**GNM, ANM and association of modes**

GNM is more robust than ANM in the prediction of residue fluctuations [[3](#_ENREF_3),[5](#_ENREF_5),[6](#_ENREF_6)]. Therefore, in this study we utilized GNM to predict the relative magnitudes of the residues’ fluctuations, to detect the hinge regions from the fluctuations and to derive the inter-residue cooperativity (or cross-correlations). The GNM analysis was computed using an in-house program with the distance cut-off set to the commonly used value of 10Å. This in house program simply implements the GNM calculations as described in previous studies [5, 6]. We relied on the contribution to the overall motion derived from the GNM analysis to identify the modes that have the largest influence on the c-ring’s movement. The analysis clearly identified five modes of motion with the largest contributions, each exceeding 1% of the overall motion, whereas the following 20 modes contributed significantly less to the overall motion (Fig. 3A). Of these five modes, two pairs of modes displayed the same eigenvalues and were identified as degenerate, and we used the average values of each such pair to obtain the residue fluctuations and cross-correlations. Overall, we analyzed three types of motion, consisting, respectively, of the averaged GNM1-2, the averaged GNM4-5, and GNM3.

In this study, we performed the ANM analysis using the HingeProt webserver [[7](#_ENREF_7)] and ANM webserver [[8](#_ENREF_8)], setting the distance cut-off to 18Å, the commonly used value, which is set as the default cut-off distance in the HingeProt webserver [[7](#_ENREF_7)]. ANM was employed to predict the directions of motion, i.e., to generate the 3D deformations that describe the three types of GNM motion. To this end, we matched the slowest ANM modes to their corresponding GNM modes. All three classes of GNM modes displayed the same hinge regions (Fig. 3B), and we relied on the inter-residue correlation matrices to match between GNM modes and ANM modes (Fig. S4). Interestingly, ANM modes 1, 2, 3 and 4, matched to motion type I (GNM1-2), consisted of the same cross-correlation matrices. The 3D deformations indicated similar extraction/contraction movement of the ring, albeit emphasized at the lumen ends at ANM1 and 2 (Movie S1), and at the stroma ends at ANM3 and ANM4 (Movie S2). Likewise, ANM modes 6-9 displayed a bending motion, and were associated with motion type III (GNM4-5).

**Comparison to normal mode analysis of other c-rings**

The GNM and ANM analysis described above was repeated for six other available c-ring structures. Analysis included GNM-derived residue fluctuations and inter-residue correlation, prediction of 3D deformations by ANM and association of the GNM modes with corresponding ANM modes (Figs. S3 and S4, Table S1). For each c-ring structure, as in the green pea c-ring analysis, the GNM analysis identified five modes that had the largest contribution to the overall motion (Fig. S3). Furthermore, as in the case of the green pea c-ring, the analysis of the other c-rings indicated that the five slowest modes consisted of two pairs of degenerate modes, resulting in a total of three main types of motion. For each of the rings, we used cross-correlation matrices to match the GNM modes to their associated ANM modes and characterized the three types of motion in the c-ring structure (Fig. S3 and Table S1). For some of the rings the order and pairing of the modes differed from those of the green pea c-ring; for instance, in the c-ring from the bovine F1-c8 sub-complex (PDB ID 2xnd), motion type I (GNM1-2 in the green pea) corresponded to GNM3, whereas motion type II (GNM3 in the green pea) corresponded to GNM1,2. Still, all three types of motion of the green pea c-ring were easily matched to the three slowest types of motion in each of the other rings, demonstrating that the activity of all available ring structures, differing in size and shape, is governed by the same slow movements.

**REFERENCES**

1. Atilgan AR, Durell SR, Jernigan RL, Demirel MC, Keskin O, et al. (2001) Anisotropy of fluctuation dynamics of proteins with an elastic network model. Biophys J 80: 505-515.

2. Bahar I, Atilgan AR, Erman B (1997) Direct evaluation of thermal fluctuations in proteins using a single-parameter harmonic potential. Fold Des 2: 173-181.

3. Bahar I, Rader AJ (2005) Coarse-grained normal mode analysis in structural biology. Curr Opin Struct Biol 15: 586-592.

4. Bahar I, Kaplan M, Jernigan RL (1997) Short-range conformational energies, secondary structure propensities, and recognition of correct sequence-structure matches. Proteins 29: 292-308.

5. Cui Q, Bahar I (2006) Normal Mode Analysis: Theory and Applications to Biological and Chemical Systems: Boca Raton : Chapman & Hall/CRC.

6. Rader AJ, Chennubhotla C, Yang L-W, Bahar I, Cui Q (2006) The Gaussian Network Model: theory and applications. Normal Mode Analysis - theory and applications to biological and chemical systems: Chapman & Hall/CRC. pp. 41-63.

7. Emekli U, Schneidman-Duhovny D, Wolfson HJ, Nussinov R, Haliloglu T (2008) HingeProt: automated prediction of hinges in protein structures. Proteins 70: 1219-1227.

8. Eyal E, Yang LW, Bahar I (2006) Anisotropic network model: systematic evaluation and a new web interface. Bioinformatics 22: 2619-2627.

9. Meier T, Polzer P, Diederichs K, Welte W, Dimroth P (2005) Structure of the rotor ring of F-Type Na+-ATPase from Ilyobacter tartaricus. Science 308: 659-662.

10. Pogoryelov D, Yildiz O, Faraldo-Gomez JD, Meier T (2009) High-resolution structure of the rotor ring of a proton-dependent ATP synthase. Nat Struct Mol Biol 16: 1068-1073.

11. Preiss L, Yildiz O, Hicks DB, Krulwich TA, Meier T (2010) A new type of proton coordination in an F(1)F(o)-ATP synthase rotor ring. PLoS Biol 8: e1000443.

12. Stock D, Leslie AG, Walker JE (1999) Molecular architecture of the rotary motor in ATP synthase. Science 286: 1700-1705.

13. Vollmar M, Schlieper D, Winn M, Buchner C, Groth G (2009) Structure of the c14 rotor ring of the proton translocating chloroplast ATP synthase. J Biol Chem 284: 18228-18235.

14. Watt IN, Montgomery MG, Runswick MJ, Leslie AG, Walker JE (2010) Bioenergetic cost of making an adenosine triphosphate molecule in animal mitochondria. Proc Natl Acad Sci U S A 107: 16823-16827.

15. Pogoryelov D, Krah A, Langer JD, Yildiz O, Faraldo-Gomez JD, et al. (2010) Microscopic rotary mechanism of ion translocation in the F(o) complex of ATP synthases. Nat Chem Biol 6: 891-899.
