# Supplementary material for: Structure and Flexibility of the C-Ring in the Electromotor of Rotary FoF1-ATPase of Pea Chloroplasts
Source: PLoS One. 2012 Sep 25;7(9):e43045. doi: 10.1371/journal.pone.0043045 (PMC3458034; doi:10.1371/journal.pone.0043045)
Supplement: Table S1 — Comparison between normal mode analyses of different c-rings. For each ring [9], [10], [11], [12], [13], [14], [15], the GNM and ANM modes corresponding to motions of types I, II and III are specified (colored green, blue and red, respectively). Modes were matched according to the ANM deformations as well as the GNM cross-correlations, as exemplified in Figure S4. (DOCX) [file pone.0043045.s006.docx]

**Table S1. Comparison between normal mode analyses of different c-rings.**

| **Structure**  **(PDB ID)** | **Monomers** | **Type I** | | **Type II** | | **Type III** | |
| --- | --- | --- | --- | --- | --- | --- | --- |
| **Green Pea c-ring** | **14** | GNM1,2 | ANM1,2,3,4 | GNM3 | ANM5 | GNM4,5 | ANM6,7,8,9 |
| **2x2v** | **13** | GNM1,2 | ANM1,2,3,4 | GNM3 | ANM9 | GNM4,5 | ANM8 |
| **2xnd** | **8** | GNM3 | ANM4,5,7,8 | GNM1,2 | ANM1 | GNM4,5 | ANM2,3 |
| **2xok** | **10** | GNM3 | ANM1,2,3,4 | GNM1,2 | ANM5 | GNM4,5 | ANM6,7,8 |
| **1yce** | **11** | GNM2,3 | ANM1,2,3,5 | GNM1 | ANM4 | GNM4,5 | ANM6,7 |
| **2w5j** | **14** | GNM1,2 | ANM1,2,3,5,8 | GNM3 | ANM4 | GNM4,5 | ANM6,7 |
| **2wie** | **15** | GNM1,2 | ANM1,2,3,4 | GNM3 | ANM5 | GNM4,5 | ANM8,9 |
| **2bl2** | **10 (4 helices)** | GNM1,2 | ANM1,2,3,4 | GNM3 | ANM16 | GNM4,5 | ANM12, 13 |
